# Ambulance Design Survey 2011: A Summary Report

**DOI:** 10.6028/jres.118.019

**Published:** 2013-10-25

**Authors:** Y Tina Lee, Deogratias Kibira, Allison Barnard Feeney, Jennifer Marshall

**Affiliations:** National Institute of Standards and Technology, Gaithersburg, MD 20899

**Keywords:** ambulance, design, emergency medical services (EMS), emergency medical technician (EMT), modeling and simulation, patient compartment, performance, safety, standard, survey

## Abstract

Current ambulance designs are ergonomically inefficient and often times unsafe for practical treatment response to medical emergencies. Thus, the patient compartment of a moving ambulance is a hazardous working environment. As a consequence, emergency medical services (EMS) workers suffer fatalities and injuries that far exceed those of the average work place in the United States. To reduce injury and mortality rates in ambulances, the Department of Homeland Security Science and Technology Directorate has teamed with the National Institute of Standards and Technology, the National Institute for Occupational Safety and Health, and BMT Designers & Planners in a joint project to produce science-based ambulance patient compartment design standards. This project will develop new crash-safety design standards and improved user-design interface guidance for patient compartments that are safer for EMS personnel and patients, and facilitate improved patient care. The project team has been working with practitioners, EMS workers’ organizations, and manufacturers to solicit needs and requirements to address related issues. This paper presents an analysis of practitioners’ concerns, needs, and requirements for improved designs elicited through the web-based survey of ambulance design, held by the National Institute of Standards and Technology. This paper also introduces the survey, analyzes the survey results, and discusses recommendations for future ambulance patient compartments design.

## 1. Introduction

Providing emergency care services in the patient compartment of a moving ambulance has proven to be a hazardous activity. EMS workers suffer fatalities and injuries that far exceed those of the average work place in the United States [1,2]. During the period between 1990 and 2009 ambulance accidents were responsible for 590 deaths and 28,989 injuries of ambulance occupants [3]. The most common injuries among emergency medical services (EMS) personnel are sprains and strains with frequency of 41 % and contusions and abrasions with frequency of 13 % [4]. Improved design and layout of the patient compartment would reduce the number of hazards inside the ambulance and ultimately save lives. However, design, construction, and performance standards that would provide a baseline for improving safety, ergonomics, and emergency medical care are currently lacking in the United States. The General Services Administration’s KKK-A-1822F standard is an ambulance procurement specification [5]. This standard covers overall construction, electrical systems, emergency warning lights, and many other aspects of ambulance design. However, unlike some foreign standards such as the Australian/New Zealand Standard 4535 [6] and British Standards Institution BS EN 1789 [7] that provide comprehensive safety specifications for the van-type ambulances in use in those countries, it does not provide such specifications for US box-type ambulances. Some states have adopted the federal KKK-A-1822F standard, while others have their own design requirements. The National Fire Protection Association (NFPA) has developed a voluntary national standard to address ambulance design for safety. This standard, code named NFPA 1917, *Standard for Automotive Ambulances* includes requirements for factors such as safety belts, seats, and access to patients, but does not address the interior layout of equipment, seating, and patients [8]. The most recent edition was published in September 2012 and took effect January 1, 2013.

To reduce the current level of occupational mortality for EMS personnel and improve patient care, it requires eliciting requirements from a wider stakeholder audience of practitioners, government, and manufacturers. The project also elicited the views of manufacturers on comparative relationships between installing optional safety features, associated cost, and mandatory design requirements.

To reduce injury and mortality rates, the Department of Homeland Security Science and Technology Directorate (DHS S&T^1^) teamed with the National Institute of Standards and Technology (NIST), the National Institute for Occupational Safety and Health (NIOSH), and BMT Designers and Planners to develop science-based ambulance patient compartment standards. This project will develop new crash-safety design standards and improved user-interface guidance resulting in the manufacture of patient compartments that 1) are safer for EMS personnel and patients, and 2) improve patient care. The methodology will include rigorous top-down requirements analysis following systems engineering practices, and requirements analyses through iterative validation of design concepts and criteria. When concluded, the project will submit validated patient compartment design requirements to the National Fire Protection Association (NFPA) for inclusion in the next edition of NFPA 1917 that is currently scheduled for publication in the summer of 2015.

**Fig. 1:**
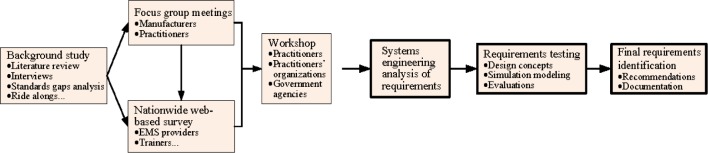
Activities to develop the patient compartment design requirements.

Previous studies in determining the optimal layout of the patient compartment have included use of optimization modeling and similar methods [9,10]. Hignett *et al.* have identified nine design challenges to improve ambulance safety using information obtained from practitioners through workshops [11]. This project was initiated with a literature review and identified the needs of the EMS community to perform a requirements analysis to support the new design of patient compartments. Figure 1 depicts the project activities. Examples of the activities include state-of-the-art technology and practice assessments followed the literature review [12], interviews with domain experts, standards review and gap analysis, ambulance ride alongs, focus group meetings with manufacturers and practitioners, and workshop with practitioners and federal stakeholders [13]. The project team has solicited needs and requirements and addressed related issues. The highlights of the first phase of the project are:

- *Focus group meetings:* to gain a broader understanding of the issues involved in ambulance safety from diverse stakeholder viewpoints.
- *Web-based survey:* to elicit information necessary to identify requirements for ambulance patient compartment design and to measure practitioner satisfaction with current patient compartment interior layout design and practice.
- *Workshop:* to establish consensus on industrial needs and requirements, and to prioritize requirements.

This paper presents an analysis of practitioners’ concerns, needs, and requirements for improved designs elicited through a web-based survey. The requirements obtained will be analyzed using systems engineering and tested by modeling and simulation [14].

The organization of the rest of this paper is as follows. In Sec. 2, an overview of the survey process is presented. Section 3 presents the survey results. Section 4 analyzes the results, discussing recommendations for patient compartment designs and patient care practices. Section 5 offers concluding remarks and recommendations for future ambulance design.

## 2. Survey Overview

The ambulance design survey was administered on the web over a period of one month – from November 29, 2011 to December 28, 2011. The respondents included a nation-wide cross-section of registered EMTs, paramedics, and emergency service organizations to elicit current practice and opinions on improvements. A total of 2537 responses were received. The survey questionnaire and details of the survey outcome are provided in the appendix of Ref. [15].

### 2.1 Introduction to the Questionnaire

The survey questionnaire contains both quantitative and qualitative questions. Most of the quantitative questions are multiple-choice questions where the respondent only needs to select one or more of the choices provided. On the other hand, qualitative questions are designed to measure the level of satisfaction with current ambulance features. The qualitative questions have a 10-point scale using the American Customer Satisfaction Index (ACSI) methodology [16]. There were also questions intended to elicit satisfaction with current designs particularly regarding work environment, ergonomics, restraint systems, and communications. With this type of questions, the survey scores are rated on a 0 to 100 scale where “0” means “poor” or “strongly disagree” and “100” being “excellent” or “strongly agree.” Each category has open-end questions to allow for respondents’ comments.

### 2.2 Question Categories

The questionnaire is grouped into eight categories:

- *Work-related information*: respondent’s affiliation with the service (professional or volunteer), type of ambulance they normally work with, and service types normally provided.
- *Seating*: type of seat, seating orientation preferred, location of seat relative to ceiling, and location of equipment and supplies.
- *Restraint systems*: type of restraint, rate of restraint use, reason for not using them, and range of mobility permitted by restraints.
- *Occupancy and transport*: capability and frequency of transporting more than one patient in their current ambulance.
- *Ergonomics*: issues related to working space, position of controls, and patient cot.
- *Communications*: availability, types, and preferred communication systems in the ambulance(s) used by the respondent.
- *Performance*: most frequently performed medical procedures; reach-ability and frequency of required equipment, supplies, and medicines use.
- *Standards*: satisfaction or expectation of current design standards.

## 3. Survey Results

This section summarizes key findings for each category in the questionnaire. The results from multiple choice questions were evaluated quantitatively and the frequency of each selection was reported as a percentage of occurrence. Respondents were also asked about their satisfaction with the current design standards in ambulances using the ACSI methodology. The scores based on this methodology are ratings on a 0 to 100 scale and not percentages. There were also open-ended comments for which the respondents provided a total of over 300 pages. Comments are in free format and without any restrictions. The comments are not trivially quantifiable, so the authors have provided an earnest attempt to synthesize those comments into a unbiased coherent overview.

### 3.1 Work Related Information

The survey results show that the majority of respondents (72 %) are career EMS providers. The majority of respondents (89 %) report that they work in box-type ambulances (Type I and Type III). The services typically performed are advanced life support (ALS) with 76 % of mentions and basic life support (BLS) with 61 %. Of a transportation time range of zero to thirty minutes, three-quarters of respondents spend between six and twenty minutes transporting patients to the hospital.

### 3.2 Seating

**Fig. 2:**
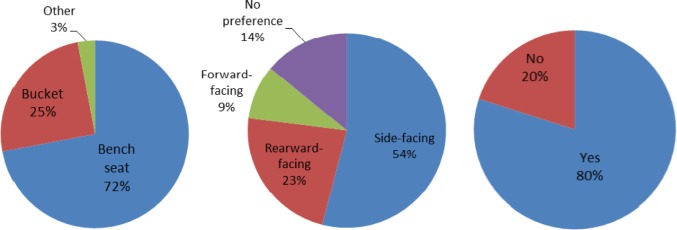
Current seating arrangement (left), seating orientation preference –both bucket and bench seat (center), and CPR seat recommendation (right).

The seating category concerns the extent to which the patient compartment will enable EMT workers to provide effective and ergonomic work from the seat in the patient compartment. The survey responses showed that the bench seat is the most commonly used seat type and side-facing is the most preferred orientation. In addition, 80 % of respondents would recommend providing a CPR seat in the patient compartment. The statistical data are summarized in Fig. 2. The results indicate that current seating arrangements are not very conducive to treating patients, nor do they provide sufficient access to equipment or supplies. When asked whether location and height of ambulance seating is adequate to providing access to patient or equipment/supplies, the satisfaction ratings were 61 and 46 respectively. This means that the respondents are slightly more satisfied with the location of the seat than the height. The satisfaction rating for seating and performing patient care is 55. This rating indicates that EMS workers have significant concerns about seating.

Through detailed review of the comments received from the respondents, the advantages and disadvantages of using bench seats verses buckets seat are synthesized and grouped into nine factors: access the patient, treat patient, allow eye contact with patient, access equipment or controls, safety in case of accident, space for monitor or jump bag, extra patient capacity, seat height, and seat orientation. Table 1 presents the comparison between the bench seat and bucket seat using the nine factors.

### 3.3 Restraint Systems

**Fig. 3:**
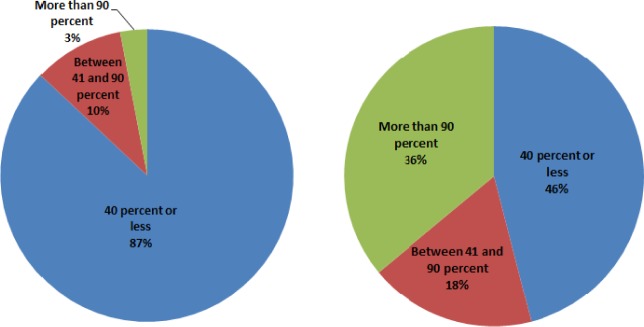
Time wearing restraint system when treating patient (left) and when not treating patient (right).

The restraint systems category concerns the extent to which the patient compartment will enable EMTs to safely and easily provide patient care and access equipment when restrained. The survey shows that lap belts are provided in 79 % of ambulances, while lap and shoulder belts are in 32 % of the ambulances. Also, 4- and 5- point restraints are in 16 % of the ambulances. Nearly half the respondents indicate that seatbelt regulations are in effect at their state or organization level. However, one-quarter of respondents do not know that such regulations exist. Figure 3 shows the percentage of the total time that EMS professionals wear restraint systems in the patient compartment when they are either treating or not treating patients.

The respondents provided reasons for not wearing restraints. The comments were assessed and the major reasons for the respondents not wearing restraints are summarized below:

- Bad habits; lack of safety culture; not required; uncomfortable
- Short transportation time
- Annoyance with having to constantly buckle and unbuckle, which are described as frustrating and inconvenient
- Self-defense, in the case of violent patients
- Do not feel safer when restrained in that current restraints offer little protection in a crash and have a potential to increase injury
- Limited the ability to safely access the patient – wearing restraint puts the EMS provider too far away from the patient or at an inconvenient orientation relative to the patient
- Unable to see and communicate with the patient
- Limits the ability to reach necessary equipment, controls, or supplies
- Unable to quickly or efficiently perform quality patient care
- Restraints not designed (e.g., not long enough) to fit all EMS workers

The satisfaction rating for restraint systems features’ capacity to allow EMS workers to do their job is 28. This result demonstrates that EMS providers are highly unsatisfied with the restraint systems. On the other hand, the awareness of the inherent risk associated with working in a moving ambulance while unrestrained is 77. Thus, EMS providers are aware of the danger but simply accept it as an inherent hazard of ambulance work. The responses indicate that if more flexible restraints that permit movement were available, EMS workers would use them.

### 3.4 Occupancy and Transport

**Fig. 4:**
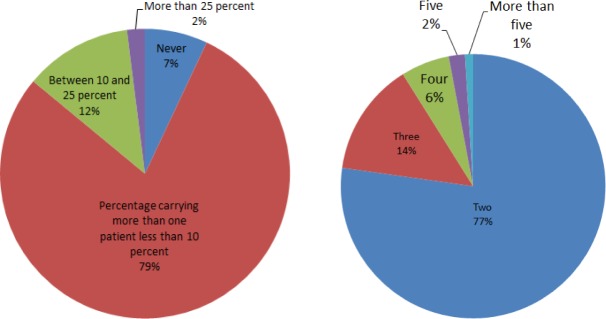
Percentage of trips that the ambulance carries more than one patient (left) and maximum number of patients that the ambulance can carry (right).

The occupancy and transport category concerns the extent to which the patient compartment will enable EMT workers to safely transport and provide patient care to more than one patient at a time. Respondents show that ambulances rarely transport more than one patient at a time. Eighty-six percent (86 %) either never transport more than one patient or have done so less than 10 % of the time. Figure 4 shows the distribution of percentage times for carrying more than one patient side-by-side with the ambulance patient transport capacity. The pie charts show that transporting one patient is generally the norm largely because of the current ambulance configuration that expects one supine patient, strapped to the cot, and receiving medical attention while in transit. A second patient in critical condition, when carried, has to be back-boarded and laid on the bench seat. However, using the bench seat for carrying a patient reduces the available seating for EMS providers as well as room to maneuver while providing the necessary medical care. Secondly, the patient on the bench seat would not be fully secured and therefore, liable to injury in case of an accident. That patient must also not be too tall; otherwise, s/he would not be able to lie down on the short stretch of the bench seat.

Respondents, particularly from rural areas, state that ambulances should be built to carry more than one patient due to poor availability of resources in those areas. However, such patients would not receive treatment while in transit because there would be insufficient space inside the patient compartment for the EMS providers to provide the necessary care.

### 3.5 Ergonomics

**Fig. 5:**
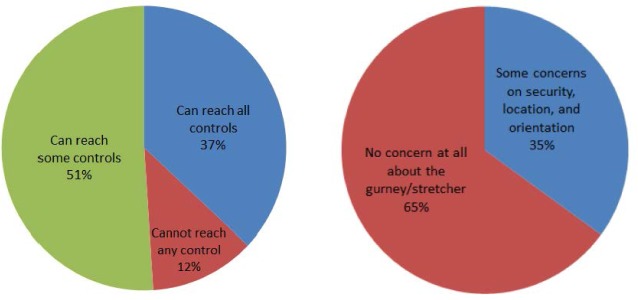
Reach of controls from seated position (left) and gurney/cot (right) concerns.

Ergonomics in the ambulance is concerned with the extent to which the patient compartment provides a task-related injury free work environment for EMT workers and patients. The survey results show that the current interior layout takes little consideration of human factors. The most frequently used equipment items such as oxygen outlets and suction units are out of reach. EMS workers, in general, have difficulty reaching controls (i.e., for lighting, HVAC systems, and radio) and have to bend over to treat patients. The observation is that placing controls on only one side of the ambulance (i.e., close to the captain’s chair) is counterproductive. Respondents recommend an additional control panel close to the bench seat where the worker most interested in using it is located. Figure 5 shows that while seated, only 37 % can reach all controls and 51 % can reach some of the controls.

The gurney/cot is the primary piece of equipment in the ambulance whose design and placement presents the most ergonomic concerns and is frequently mentioned in respondents’ qualitative comments. Most respondents are comfortable with the current gurney/cot design; but, most think that it should be located in the center rather than being close to the passenger side. The elevation of the patient should also be increased, as should the space around the gurney/cot to improve mobility necessary for the patient treatment. The gurney/cot position should be adjustable along more axes for better access while the locking mechanisms to the floor are considered cumbersome to use. Consideration should also be made to have the patient face forward for better comfort during transportation.

The satisfaction rating for sufficient room and general mobility around the patient compartment, when caring for patients, is moderately satisfactory at 64 while the rating for ergonomic features is a less reassuring 50. We conclude that ergonomics of the ambulance patient compartment could be significantly improved.

### 3.6 Communications

The communication category concerns the extent to which the patient compartment can 1) enable efficient and effective communications within and between the patient compartment and driver, and others, 2) facilitate driver awareness of activity in the patient compartment, and 3) facilitate the EMS worker’s awareness of driver actions. While scores for the need of effective communications systems are not as high as for the other factors, respondents recognize the role of communications in facilitating their job. To communicate between the driver and the EMS workers, most respondents (54 %) report that they mainly use verbal means. Radio communications are used with a frequency of 9 %. The wireless headsets and intercom are also in use. About half of the respondents feel that verbal communication, including yelling, is the most effective. The main reason given is that this form of communication does not disrupt the work of either the driver or the EMS providers. Some would prefer a speaker since it has the capacity to overcome the noisy work environment.

Of radio communications, the mobile radio transceiver is more frequently used than the portable radio, although over one-third cannot reach it without getting up. The portable radio, while used slightly less frequently than the mobile, is much more accessible with nearly 75 % reaching it without strain. The respondents also observe that systems such as handheld radios have a tendency to disrupt patient care while in use. These systems may also affect interaction between the EMS provider and the patient. In some cases, the EMS worker is wearing personal protection equipment (PPE) that may be contaminated, necessitating removal before using the handset. Visual displays are used in 1 % of cases. The satisfaction rating is 63 indicating that respondents feel somewhat positive that the current communication systems allow them to do their job.

### 3.7 Performance

The performance category concerns the extent to which the patient compartment will facilitate accessibility of frequently used equipment and supply. This section discusses the results from responses to questions on frequency of use and the ease of access to equipment and medicines for treating patients. The survey provided a categorized listing of equipment commonly found in an ambulance patient compartment. The categories are diagnostic equipment, ventilation/respiration equipment, infusion material and equipment, or equipment used for managing life-threatening situations.

#### 3.7.1 Diagnostic Equipment

Stethoscopes and blood pressure monitors are the most used diagnostic equipment. Nearly all respondents (96 %) use a stethoscope a majority of the time and nearly as many use the blood pressure monitor (95 %). Other equipment used includes oximeter (88 %), blood glucose meter (68 %), and diagnostic light (59 %). Regarding reach, stethoscopes are relatively within reach since 77 % can reach them from the seated position without strain while the blood pressure monitors are more difficult with 57 % able to reach them without strain. The oximeter is also relatively difficult to reach since one-quarter (26 %) need to get out of their seats to reach it and half (51 %) can reach it from the seated position without strain. The blood glucose meter was difficult to reach with one-third (32 %) having to leave their seats. The most difficult to reach diagnostic equipment is the thermometer where 41 % of respondents have to leave their seat to reach it; however, it is infrequently used. Diagnostic equipment, other than mentioned above, are reported to never have been used at all by 82 % of the respondents. These results show that the blood pressure monitor, oximeter, and blood glucose meter need to be moved closer to the seated EMS workers than their current usual locations.

#### 3.7.2 Ventilation/Respiration Equipment

Stationary oxygen is used a majority of the time by 81 % of respondents, while portable oxygen is used a majority of the time by 65 % of respondents. Most of the other ventilation/respiration equipment is used less frequently. Regarding reach, oxygen outlets are the most difficult to reach. Half of the respondents need to leave their seat to reach the portable oxygen while only 32 % can reach it without straining. Less than half need to leave their seats (40 %) to access the stationary oxygen. The rest of the equipment is still difficult to reach with 68 % unable to do so while seated. These results show that locating oxygen outlets closer to the seated position of the EMS worker will improve performance.

#### 3.7.3 Infusion Material and Equipment

Infusion solutions and the associated equipment are used a majority of the time by slightly more than half of the respondents. Infusion mounting points are accessed with frequency of 43 %. In terms of reach, only one-fifth can reach infusion solutions without strain and 58 % need to leave their seat. For injections, 55 % need to leave their seat to access them. The most difficult-to-reach equipment is the infusion system for administration of warm fluid since 71 % are unable to reach them from a seated position. However, it is rarely used since 51 % of respondents report never to have used it. Therefore, the results show that equipment for injections and infusions and their mounting points need to be put in closer proximity to the EMS provider.

#### 3.7.4 Equipment for Managing Life-Threatening Situations

In general, most of the equipment for managing life-threatening situations is used less frequently than other equipment. Cardiac monitors are used a majority of the time by 69 % of respondents. The next-most-used equipment is the defibrillator, with 41 % frequency of use. The frequencies of use for nebulization apparatus, capnometer, and external cardiac pacing are 23 %, 20 %, and 20 % respectively. The least-used equipment is the central vein catheters. The cardiac monitor and the defibrillator can be reached by 37 % and 41 % of respondents respectively without leaving the seated position. Rarely-used equipment for managing life-threatening situations is also mentioned as most unreachable.

#### 3.7.5 Supplies and Personal Protective Equipment

The most commonly used supplies include non-sterile gloves for single use, sharps container, and blankets that are used by 83 %, 69 %, and 67 % respectively, a majority of the time. Less frequently used supplies include materials for treatment of wounds, vomiting bag, kidney bowl, and sterile surgical gloves. The hazmat suit is least used since a majority (52 %) report never to have used it. The frequently used sharps container is the only item that can be reached without strain by a majority of respondents (56 %). The percentages of EMS workers who have to leave their seats to reach blankets and non-sterile gloves for single use are 59 % and 35 % respectively. The corresponding percentage for sharps containers is 19 %.

#### 3.7.6 Medicine Storage

The jump bag is used a majority of the time by 78 % of respondents, while locked narcotics are only used a majority of the time by 25 % of respondents. Despite its frequent use, only 27 % can access the jump bag without strain and nearly half (49 %) of respondents cannot reach the jump bag without leaving their seat. Locked narcotics are only accessible without strain to 17 % of EMS workers. These results show that jump bags should be secured in close proximity to the EMS provider seat.

### 3.8 Standards

The respondents were asked about their level of satisfaction with the current design standards for ambulances. The ACSI asks three questions, which include overall satisfaction, satisfaction compared to expectations, and satisfaction compared to the ideal. Scores indicate that EMS workers are quite unsatisfied with the current design standards with the satisfaction rate at only 49.

## 4. Discussion of Survey Results

### 4.1 Analysis

#### 4.1.1 Seating

The design of seats and how seating impacts emergency service provision is a hot topic in the EMS community. The focus of the survey addresses the issues related to safety, performance, and ergonomic concerns of seating types (bucket seat, bench seat, or CPR seat), orientation of seat (side-facing, rear-facing, or forward-facing), and seat configuration (options to move forward/backward, change the orientation, adjust seat height, etc.). However, there is no one-size-fits-all solution and each type/feature has advantages and disadvantages.

The survey shows that the most commonly used seat type and orientation in today’s ambulances is the bench seat (72 %) with side-facing orientation (54 %). However, the survey results do not necessarily imply bench seat and side facing are the respondents’ preference. The primary concerns related to seating are 1) reachability of the patient, equipment, and supplies, and 2) the flexibility of seat configuration (e.g., adjustment of height, forward-backward moving, or orientation). The seat features and functions need to be enhanced for better patient care and ergonomics. Different patient-care tasks and EMT stature affect the reachability. Thus, the location and height of the seat are important to EMTs. With higher flexibility of seat configuration, EMT workers will have better access to the patient and equipment, and better interaction with the patient.

#### 4.1.2 Restraint Systems

The inability of EMS workers to remain safely restrained, while treating patients in the patient compartment of a moving ambulance, has been identified as a key impediment to EMS worker safety in North America. NIOSH examined the performance of two-, four-, and five-point restraints, especially the biomechanical and kinematic effects on EMS workers in simulated and actual ambulance patient compartments during crash events. The results indicate that the inclusion of restraint systems that offer mobility have the potential to improve worker safety under many working conditions [17].

The survey shows lap belts (79 %) are commonly provided in today’s ambulances. However, it is not a common practice for the EMS workers to wear their restraint systems. Most EMS workers contend that it is very challenging to reach for equipment and supplies and care for patients while remaining restrained. For example, 36 % of respondents wear their restraint systems most of time when not treating patients while only 3 % wear restraint systems most of time when treating patients. The respondents’ concerns on current restraint systems are the systems’ capacity to enable mobility, ergonomics, and safety functions. The design of restraint systems has to be improved for safety, efficiency, and comfort. For example, offering different types of restraint systems according to specific needs (e.g., an advanced system for allowing perform tasks while standing) will help EMT workers secure more safely and more comfortably, and hence perform tasks more efficiently.

Most serious injuries occur in the rear of an ambulance from improperly restrained or unrestrained occupants. The main reasons for not wearing restraint systems are habit and work culture. It is important for EMS workers to change their attitude toward seat belts. Well-enforced regulations and education will be another mechanism to increase restraint use. Also, the ability to reach the patient is just as important as the ability to reach equipment. Effectively secure people and equipment in the patient compartment enables safe treatment in transit for EMS workers (seated or standing) and patients (seated or on stretchers).

#### 4.1.3 Occupancy and Transport

The current design of the ambulance seems to be suitable for carrying only one patient in critical condition and up to two others or family members riding as ordinary passengers. The inability to maneuver while carrying multiple patients and an even greater concern for safety of patients and crew are the main reasons for ambulances’ inability to carry multiple patient who need ALS.

The design and layout of the ambulance determines the number of patients that can be transported. Whereas ambulances typically carry one patient at a time, the capability to carry more than one patient would be especially useful in the case of mass casualties. However, the decision to carry more than one patient depends on the condition of the patients. For the case of “walking wounded” or “non-critical” cases, it is possible to carry up to four patients – one on the gurney and three seated – accompanied by one EMT, but, with the arrangement that no medical care is to be provided during transit.

The more patients are carried the less room is available for EMS workers. As a consequence, some EMS providers think that more ambulances are needed to enable carrying multiple patients. One of the ideas fronted in the survey to avoid increasing ambulance size is installing bucket seats that can be folded to form a flat surface for the second patient in case the ambulance does not have a bench seat. Other ideas include installing suspended stretchers such as those used in the military.

#### 4.1.4 Ergonomics

An ergonomic patient compartment design should take into account the effect of the work environment on the human body so that there are no long-term musculoskeletal injuries. Survey responses indicate that installing of state-of-the-art features in seating and gurney design would greatly improve ergonomics. For example, it was mentioned that the bench seat should be replaced with individual seats that are more ergonomically designed. There is also an observation that current seats offer no lumbar support, which is necessary for a healthy back. Third, the ceiling height is low, which is a problem for some EMS workers, especially those more than six feet tall; and, there are many such EMS workers. The last example concerns steps into and out of the ambulance that are also much taller than a standard step making ingress and egress difficult.

The respondents recognize that the newest ambulances have some of the desired features such as double control panels, better securement of equipment, and more space for movement around the gurney/cot. The general observation from the survey is that ergonomics is largely not considered in most current designs. There is much stretching and turning of the human body during the course of the work that can lead to long-term health problems indicating a call for improvements. However, manufacturers’ experience is that each customer is interested in a different design with specialized features. Such an experience has to be balanced against a requirement to design an interior layout where all items can be within arm’s reach of a seated and/or restrained EMS worker.

#### 4.1.5 Communications

It is necessary to maintain communication between the driver and the EMS providers in the patient compartment, and between the ambulance and third parties such as the hospital and dispatcher. In some emergency situations, doctors at the hospital may have to provide instructions to EMS providers on procedures to enhance care and possibly save the life of the patient. On the other hand, the EMS workers often need to be informed of impending driver actions such as braking hard or making a sudden or sharp turn. In this respect, a number of communication methods are used including verbal, handheld radios, and headphones. Computers are also used in the ambulances mainly for computer-aided dispatch, patient-care reporting, charting, messaging, global positioning (navigation), and as data terminals.

The majority of respondents showed that verbal communication is widely used; probably because it is the only readily available option. Most of the other systems require using one’s hand(s) thereby interfering with medical care provision. Respondents think that what would be required are systems that do not disrupt the driver and EMS activities. Such systems would probably be integrated with head protection and not interfere with the communication between the EMS provider and the patient. Noise, particularly due to the diesel engine that is used in most ambulances, is a too often neglected factor in patient compartments since it affects the clarity of communicated messages. The required communication systems should overcome this noise. Installing soundproofing materials in the walls and floor would further enhance communication by keeping noise away from the compartment.

#### 4.1.6 Performance

The purpose of this performance section of the survey was to determine which equipment is used more frequently in current ambulances. The survey results will serve as a guide in determining the placement of equipment, medicine and supplies in relation to the seated EMS workers. Safety and performance of clinical care depends largely on easy access to equipment, medicine and supplies. Inevitably, some equipment is used more frequently than others. Examples of frequently used equipment/supplies are oxygen cylinders, stethoscope, monitors, infusion equipment, sterile gloves, and jump bag. The equipment most often used should be kept as close to the seated EMS worker as possible and clamped so that it does not dislodge during transit.

### 4.2 Recommendations

Through the analysis of the outcome of the focus group meetings, literature reviews, and the survey, we have identified the following design needs.

#### 4.2.1 Seating

The ambulance seats, while satisfying the needs of EMTs from diverse populations, should allow an EMS provider to:

- easily see and use a seat belt, which is available for each seat
- easily access or use monitors, equipment, supplies, as well as switches and controls while remaining seated
- easily access the patient
- perform all patient care tasks safely and ergonomically while remaining seated
- adjust seat positioning to face and interact with the patient
- set a child or infant patient in the child or infant seat that is restrained and oriented in a forward facing direction (for ambulances that provide a child/infant seat)

#### 4.2.2 Work Environment

The ambulance work environment should provide:

- sufficient working space around the patient cot for supporting patient care tasks safely and ergonomically
- appropriate interior height with appropriate flooring for the riders’ safety and comfort
- safety mechanisms such as padding, hand holds, and grab bars
- safe and easy ingress and egress to and from the ambulance
- surfaces that are free of sharp edges, corners, and projections
- mount hangers, hooks, or supports that are safe and easy to access
- an acceptable indoor comfort zone and interior sound level
- sufficient interior lighting to illuminate the primary patient care areas, storage areas, shelves, and floor
- automatically activated lighting when the patient compartment door is opened
- a patient cot retention system for use with any standard cot that is equipped with restraints
- securely stored equipment, supplies, and monitors while keeping them accessible
- securely fastened equipment, supplies, or monitors while using them during vehicle motion
- securely stored patient and EMT belongings
- secure and accessible receptacles for general waste and also for sharps disposal

#### 4.2.3 Restraint Systems

The ambulance restraint systems while satisfying the needs of EMTs of various statures should allow an EMS worker to:

- easily buckle and unbuckle the restraint system, which is provided at each working position
- access the patient while remaining restrained
- have face to face interaction with the patient while remaining restrained
- access or use monitors, equipment, supplies, as well as switches and controls while remaining restrained
- perform patient care safely and ergonomically while remaining restrained

#### 4.2.4 Common and Critical Equipment

The ambulance compartment provides:

- appropriate communication between the patient compartment and the driver’s compartment or third parties
- devices specifically designed for holding and securing an IV bag or bottle
- an oxygen system capable of storing and supplying required medical oxygen
- an electrically powered suction aspirator system capable of providing required airflow and performing required vacuum levels

## 5. Conclusions

This paper has presented and discussed the results of a survey on ambulance patient compartment design. The questionnaire used was based on identified factors such as seating, restraint systems, occupancy, communications, and ergonomics that are relevant to safety and performance. For seating, we conclude that seats that can be adjusted to slide along three axes and rotated to face any direction while being able to be configured to carry a second back boarded patient would be most preferable. Regarding restraints, the most preferred systems should permit the EMS provider more movement and be easy to buckle and unbuckle. Communication is one of the most important factors in the patient compartment, but current systems, except yelling, have a tendency to interfere with emergency care work. Therefore, we conclude the preferred systems should not disrupt communication between the EMS provider and the patient; and should not be impeded by noise in the compartment. Ergonomics, largely ignored in ambulance layout, should be a major concern. Designs should employ best practices in the selection of seats, restraints, compartment dimensions, cabinets, equipment placement, and communications. Although patient compartment design is a major determinant of safety and task performance, emerging technologies, which when adopted for emergency medical care, can also be a major factor. Examples include hands-free communication systems, biomedical telemetry, and compact monitors that can better meet many critical safety and performance needs. While such technologies are not considered in the current study, they will no doubt play a role in determining what systems installations to recommend as standard features in future ambulances.

The importance of this survey to the EMS community can be manifested by the large number of responses; i.e., 2537 over the one month period. This activity was preceded by high caliber attendance and vigorous responses and inputs during focus group meetings, individual meetings, ride alongs and later, the workshop. Respondents’ comments including viewpoints, concerns, needs, preferences, and recommendations exceeded 300 pages and are still being analyzed and synthesized.

The next steps in the project include prioritizing, updating, and finalizing a preliminary set of design requirements. Based upon which, the design criteria will be reviewed to reflect the new set of requirements. Design concepts based on the design criteria will be developed and modeled using computer-aided-design systems. Within a work environment based on these models, EMT performance of ambulance patient care activities will be simulated to validate the design requirements. Various design concepts will then be developed iteratively in response to challenges of task performance feasibility and safety. The conclusion of this activity will generate a final set of design requirements that will form the basis for recommendations for inclusion into the next version of the NFPA 1917 standard.

Besides insufficient ambulance design standards that are being addressed by this research, many EMS workers acknowledge other prevailing safety challenges. Among them is a wrong attitude towards safety in the ambulance work environment. EMS practitioners readily accept patient compartment hazards as an inherent risk associated with emergency medical care. For example, the research has shown that most EMS workers do not wear seat belts even when they are not treating patients. This attitude needs to be changed by instilling a culture of workplace safety within the emergency care practice through training, legislation, and regular inspections and drills. Another issue to be addressed is the range of clinical procedures performed on patients during transit to the hospital. A structured decision-making approach could be used to determine the procedures that need to be performed at the site before the patient is carried onto the ambulance. Instituting best practices in the delivery of emergency medical care is another dimension along which safety challenges could be addressed. It may well be that some medical care activities routinely performed while the ambulance is in motion, thus putting EMS workers’ safety in jeopardy, are not absolutely necessary. A study addressing clinical care activities, work culture, regulations, training, and work practice in addition to ambulance design would more comprehensively enhance emergency patient care and safety.

## Figures and Tables

**Table 1:** Bench Seat and Bucket Seat Comparison

Seat type	Bench seats	Bucket seats
Factor		
Access the patient	Easier	Harder
Treat patient	Generally easy; easy for IV, not easy for airway or CPR	Generally difficult; easy for airway
Allow eye contact with patient	Yes	Limited
Access equipment or controls	Limited	Easier
Safety in case of accident	This is a challenge; can be improved using safety nets	Forward facing improves safety
Space for monitor or jump bag	Yes	No
Extra patient capacity	Possible	No
Seat height	Fixed	Adjustable
Seat orientation	Side facing	Forward, rearward, and/or side facing

## Footnotes

1 DHS’ Science and Technology Directorate (S&T) Resilient Systems Division (formerly the Human Factors/Behavioral Sciences Division) and First Responders Group.
